# CCNI2 promotes the progression of human gastric cancer through HDGF

**DOI:** 10.1186/s12935-021-02352-6

**Published:** 2021-12-11

**Authors:** Wenchao Chen, Yang Zhou, Gang Wu, Peichun Sun

**Affiliations:** Department of Gastrointestinal Surgery, Henan Provincial People’s Hospital, Zhengzhou University People’s Hospital, Henan University People’s Hospital, Zhengzhou, 450003 Henan China

**Keywords:** Gastric cancer, CCNI2, Proliferation, Apoptosis, Migration, RNA sequencing, HDGF

## Abstract

**Background:**

Gastric cancer is a highly aggressive malignant tumor with heterogeneity and is still a global health problem. The present study aimed to investigate the role of Cyclin I-like (CCNI2) in the regulation of phenotype and tumorigenesis, as well as its underlying mechanisms.

**Method:**

The expression profile of CCNI2 in gastric cancer was determined based on The Cancer Genome Atlas (TCGA) database and immunohistochemical staining. The effects of altered CCNI2 expression on the biological phenotypes such as proliferation, clone formation, apoptosis and migration of gastric cancer cell lines BGC-823 and SGC-7901 were investigated. Mice xenograft models were established to reveal the role of CCNI2 knockdown on tumorigenesis. The potential mechanism of CCNI2 regulating gastric cancer was preliminarily determined by RNA sequencing.

**Result:**

CCNI2 was abundantly expressed in gastric cancer and was positively correlated with pathological stage. Knockdown of CCNI2 slowed down the malignant progression of gastric cancer by inhibiting tumor cell proliferation, increasing the susceptibility to apoptosis and suppressing migration. Moreover, downregulation of CCNI2 attenuated the ability of gastric cancer cells to form tumors in mice. Additionally, there was an interaction between CCNI2 and transcription factor hepatoma-derived growth factor (HDGF) in SGC-7901 cells. Knockdown of CCNI2 alleviated the promoting effects of HDGF overexpression in gastric cancer cells.

**Conclusions:**

CCNI2 promoted the progression of human gastric cancer through HDGF, which drew further interest regarding its clinical application as a potential therapeutic target.

**Supplementary Information:**

The online version contains supplementary material available at 10.1186/s12935-021-02352-6.

## Background

Gastric cancer is a highly aggressive malignant tumor with heterogeneity and is still a global health problem [1]. Current statistics show that gastric cancer as the fifth most common cancer and the third most common cause of cancer death in the world [2]. In addition, the incidence of gastric cancer increased progressively with the age [3]. Unfortunately, most patients with gastric cancer are diagnosed at an advanced stage, and treatment is often ineffective [4]. To date, gastrectomy and chemotherapy are the mainly therapeutic options for gastric cancer patients, but drug resistance, either acquired or primary, is the main cause for treatment failure [5]. In view of the fact that cancer is a multi-stage disease process, characterized by the gradual development of various gene expression mutations and epigenetic alterations, precise targeted therapy has become a hotspot. Recently, targeted therapies licensed to treat gastric cancer include trastuzumab (HER2-positive patients first line), ramucirumab (anti-angiogenic second line), and nivolumab or pembrolizumab (anti-PD-1 third line) [6–9]. The heterogeneity and drug resistance of patients with gastric cancer are still key obstacles to targeted therapy. Therefore, a comprehensive understanding of the mechanisms of gastric cancer is required to overcome these challenges.

Cyclin-dependent kinase 5 (CDK5) exerts important roles in gene expression, cytoskeleton dynamics, signal cascade, and cell survival in a variety of cells [10, 11]. The kinase activity of CDK5 is tightly regulated by specific activators including p35, p39, and cyclin I (CCNI). As a homolog of CCNI, CCNI2 interacts with CDK5 and activates the kinase activity of CDK5 to participate in cell cycle regulation [12]. In addition, tumor-associated cell cycle defects are often mediated by alterations in CDK5 activity. Abnormal overexpression of CDK5 can lead to genome and chromosome instability as well as accelerate cell proliferation [13]. As a result, the dysregulation of CDK5 has been implicated in various diseases, such as Alzheimer’s disease (AD), amyotrophic lateral sclerosis (ALS), Parkinson’s disease (PD) and cancers [10, 11, 14]. Thus, inhibition of CDK5 activity may have therapeutic effects on some human tumors [15]. Moreover, Cyclin I-like (CCNI2), a homologue of CCNI, is a novel CDK5 activator. It reported that cell cycle progression and proliferation were inhibited after knockdown of CCNI2 [16]. Recently, Lai et al., demonstrated that CCNI2 played a promoting role in the progression of colorectal cancer [17]. Accordingly, these studies point towards a potential promoting effect of CCNI2 on cancers that warrants further investigations.

Therefore, the present study investigated the role of CCNI2 in the regulation of gastric cancer on proliferation, apoptosis, migration and tumorigenesis, as well as its underlying mechanisms. In this study, CCNI2 expression levels in gastric cancer were predicted in the database and further confirmed by immunohistochemical staining. In vitro and in vivo loss-of-function assays were performed in BGC-823 and SGC-7901 cells [18] to explore the role of CCNI2 in gastric cancer. Moreover, the potential downstream mechanism of CCNI2 regulating gastric cancer was initially identified through gene microarray. Altogether, our data revealed the promotive role of CCNI2 in the progression of gastric cancer and drew further interest regarding its clinical application as a potential therapeutic target.

## Methods

### Tissue microarray (TMA) and immunohistochemical staining

The protocol was approved by the Institutional Ethics Committee of the Henan Provincial People’s Hospital, and informed consent of all patients was obtained. The pathological specimens were collected from 150 patients with gastric cancer who underwent primary surgical resection. The patients with gastric cancer treated surgically only without co-morbidities were included in the study. Notably, normal tissues used in the TMA were collected from paracancerous tissues of gastric cancer patients. TMA sections contained tumor tissues (n = 150) and matched normal tissues (n = 150), which were constructed according to the methods in the literature [19]. Formalin-fixed paraffin-embedded samples were cut into 5-µm sections and deparaffinized and rehydrated. Following the manufacturer’s protocols, the sections were treated with diluted primary antibody against CCNI2 (1:50, Thermo Fisher Scientific, PA5-35081) at 4 °C overnight and secondary antibody HRP goat anti-rabbit IgG (1:200, Beyotime, A0208) at room temperature for 30 min. subsequently, sections were stained by DAB and hematoxylin at room temperature to detect the signal. The intensity of CCNI2 positive staining in TMA sections was scored as previously describe [20]. Cases showing hybrid scores of more than or equal to the median were considered as CCNI2 high expression.

### Cell culture condition

Human gastric cancer cell lines, BGC-823 and SGC-7901 were purchased from Cell Bank of the Chinese Academy of Sciences (Shanghai, China), where the cell lines were authenticated by STR profiling before experiment. The cell lines were maintained in Dulbecco’s modified eagle medium (DMEM) (Gibco, Thermo Fisher Scientific, USA) supplemented with 10% fetal bovine serum (Gibco), 100 units/mL penicillin, and 100 mg/mL streptomycin in a 5% CO_2_ humidified incubator at 37 °C.

### RNA interference and lentivirus transfection

Small RNA interference specifically targeted human CCNI2 (shCCNI2) and non-specific negative control (shCtrl) was synthesized. Lentiviral vectors with green fluorescent protein (GFP) labels were purchased from Shanghai Bioscienceres (Shanghai, China). Lentivirus plasmid including shCtrl (control plasmid), shCCNI2 (CCNI2 knockdown recombinant plasmid), and HDGF (amplified lentiviral plasmid) was constructed using T4 DNA ligase (Gibco, Thermo Fisher Scientific, USA) following the manufacturer’s protocols, respectively. BGC-823 and SGC-7901 cells were cultured for 24 h and transfected with recombinant lentivirus using Lipofectamine® 2000 (Invitrogen; Thermo Fisher Scientific, USA) at 10 MOI (multiplicity of infection). The sequence is as follows: shCCNI2-1: 5′-TACCTGCATTGCGCCACAATT-3′, shCCNI2-2: 5′-ATCTGCGACGCCTTCGAGGAA-3′, shCCNI2-3: 5’-CCTGGAAGGCGACCTGGACGA-3′.

HDGF-F: 5′-GATTCTAGAGCTAGCGAATTCGCCACCATGCACCCGGAAGGTGGCCAATTTG-3′, HDGF-R: 5′-TCCTTGTAGTCCATACCGGTCAGGCTCTCATGATCTCTGATGCC-3′.

### RNA extraction and qPCR

Total RNA isolation from BGC-823 and SGC-7901 cell lines using Trizol reagent (Invitrogen, Carlsbad, CA, USA) following the manufacturer’s protocols. The qPCR procedures were performed using the SYBR Green master mix (Thermo Fisher Scientific) as previously describe [21]. The mRNA expression of CCNI2 and HDGF was assessed by threshold cycle CT values and analyzed using 2^ΔΔCt^ method. All samples were performed in triplicate three times and the GAPDH mean value was used to normalize gene expression. The primers for amplification of human genes were listed as Additional file 1: Table S1.

### Protein extraction and western blot analysis

Preparation of whole cell lysates with protease inhibitor cocktail and phosphatase inhibitor cocktail (Roche, Alameda, CA, USA). Protein concentration was determined through BCA protein assay kit (Beyotime, Jiangsu, China). Equal amounts of protein were subjected to 10% SDS-polyacrylamide gel electrophoresis, transferred to PVDF membranes (Millipore, Danvers, MA, USA) and hybridized with corresponding primary antibody (Additional file 1: Table S2) overnight at 4 °C. The PVDF membranes were washed with TBST three times and incubated with HRP-conjugated secondary antibody for 2 h at room temperature. Signals were visualized by chemiluminescence ECL kit (Thermo Fisher Scientific) and Odyssey Infrared scanning system (Li-Cor, Lincoln, NE, USA).

### Co-immunoprecipitation (Co-IP) assay

A target protein-specific diluted primary antibody against CCNI2 or HDGF (Additional file 1: Table S2) in conjunction with protein A/G affinity beads (Santa Cruz Biotechnology) for 1 h at 4 °C. The bead-antibody complex was suspended with protein lysate and washed with extraction buffer for 3 times. Subsequently, the immunoprecipitants were subjected to western blot.

### Cell proliferation assays

BGC-823 and SGC-7901 cells were cultured in 96-well plates at a density of 2000 cells/well. After Methyl thiazolyl tetrazolium (MTT) was added to the well, the OD490 value was detected under a microplate reader (Thermo Fisher Scientific) at 1-, 2-, 3-, 4- and 5-day post cell seeding, and the growth curve was drawn to analyze the cell proliferation ability. Moreover, Celigo cell counting assay was performed to determine cell proliferation ability. The cells with GFP were identified with Celigo (Nexcelom), photographed, counted at predetermined time and the cell growth curve was plotted.

### Colony formation assay

BGC-823 and SGC-7901 cells were cultured 14 days in six-well plates at a density of 1000 cells per well. Cell colonies were washed with cold phosphate buffer brine (PBS) for 2 times, fixed with 75% ethanol, stained with 0.1% crystal violet, counted and photographed.

### Cell apoptosis detection

BGC-823 and SGC-7901 cells were inoculated into six-well plates (2 mL/well) cultured for 5 days. Following the manufacturer’s protocols of FITC (fluorescein isothiocyanate) Annexin V apoptosis detection kit I (BD Biosciences), the cell precipitation was successively eluted with precooled D-hanks (pH=7.2 ~ 7.4) and 1 × binding buffer. Cell precipitates were resuspended with 200 µL 1 × Binding Buffer, stained with 10 µL Annexin V-APC at room temperature for 15 min and cell apoptosis was detected by flow cytometry on a BDTM LSR II (BD Biosciences) equipped with FlowJo software (version vx 0.7) (BD Biosciences).

### Transwell assay

Transwell chambers (24-well, 8-mm pore, Corning, MA, USA) were performed to estimate the BGC-823 and SGC-7901 cells migration ability in 24-well plates. The cells were digested by trypsin, resuspended into cell suspension (80,000 cells/well) and placed into Transwell chambers cultured for 72 h. The medium was removed from the upper and lower compartments and 500 mL of 70% ethanol was placed in the lower compartment to fix the cells at room temperature for 15 min. The non-invading cells on the upper chamber were removed, while the cells adhering to the Polycarbonate membrane was fixed with 4% precooled paraformaldehyde for 30 min and stained with 0.5% crystal violet for 30 min at room temperature. Finally, the migrated cells were photographed from five randomly selected fields under a 200× microscope.

### Wound-healing assay

BGC-823 and SGC-7901 cells were cultured into 6-well plates (100 µL/well) at a density of 4000 cells per well. The experimental procedures were performed as described previously [22]. Cells were washed with PBS, fixed with 3.7% paraformaldehyde (Corning) for 15 min, stained with 1% crystal violet (Corning) for 10 min and viewed under a microscope for image acquisition. Cell migration distance (µm) at 0 h, and 48 h was quantified through Image J software (National Institutes of Health).

### Mice xenograft tumor assay

The experimental procedures performed on the mice were approved by the Ethics Committee of Henan Provincial People’s Hospital and accordance with Guide for Care and Use of Laboratory animals (NIH publication number 85-23, revised at 1996). BALB/c nude specific pathogen free (SPF) mice (4 weeks, 18 to 23 g; Lingchang biological, Shanghai) were kept in a sterile environment and fed normally. SGC-7901 cells transfected with lentivirus were digested with trypsin, injected into the right forearm of mice (500 µL, 6 × 10^6^ cells/mouse) and divided into shCtrl (n = 10) and shCCNI2 (n = 10) groups. After mice were injected with tumor cells for 7 days, the tumor volume was monitored 1 to 2 times a week and calculated as follows: π/6 × L×W × W (L represented long diameter and W represented short diameter). After 26 days, the mice were anesthetized with 0.7% sodium pentobarbital (10 µL/g) and evaluated the intensity of tumor fluorescence imaging under the IVIS spectral imaging system (emission wavelength of 510 nm). Subsequently, the mice were sacrificed by cervical dislocation, tumors were excised and weighed. Afterwards, the mice tumor tissues were subjected to immunohistochemical staining to evaluate Ki67 (1:200, Abcam, ab16667) expression levels.

### RNA sequencing

RNA was purified from SGC-7901 cells transfected with shCtrl/shCCNI2 and sequenced using Affymetrix Prime View human gene chip (Affymetrix Scanner 3000 scan). According to the criteria of |Fold Change| ≥ 1.8 and false discovery rate (FDR) ≤ 0.05, differentially expressed genes (DEGs) were identified, and hierarchical clustering was performed. The interaction network between CCNI2 and DEGs was further analyzed based on Ingenuity Pathway Analysis (IPA).

#### Statistical analysis

Data were obtained from three independent experiments which were presented as the means ± standard deviation (SD) and P value < 0.05 was considered significant. Comparisons between different groups were analyzed with unpaired Student’s *t* test. The significance of differences between groups was assessed by GraphPad Prism V8.0 (GraphPad, CA, USA) and SPSS 20.0 (IBM, SPSS, IL, USA).

## Results

### CCNI2 is abundantly expressed in gastric cancer

**Fig. 1:**
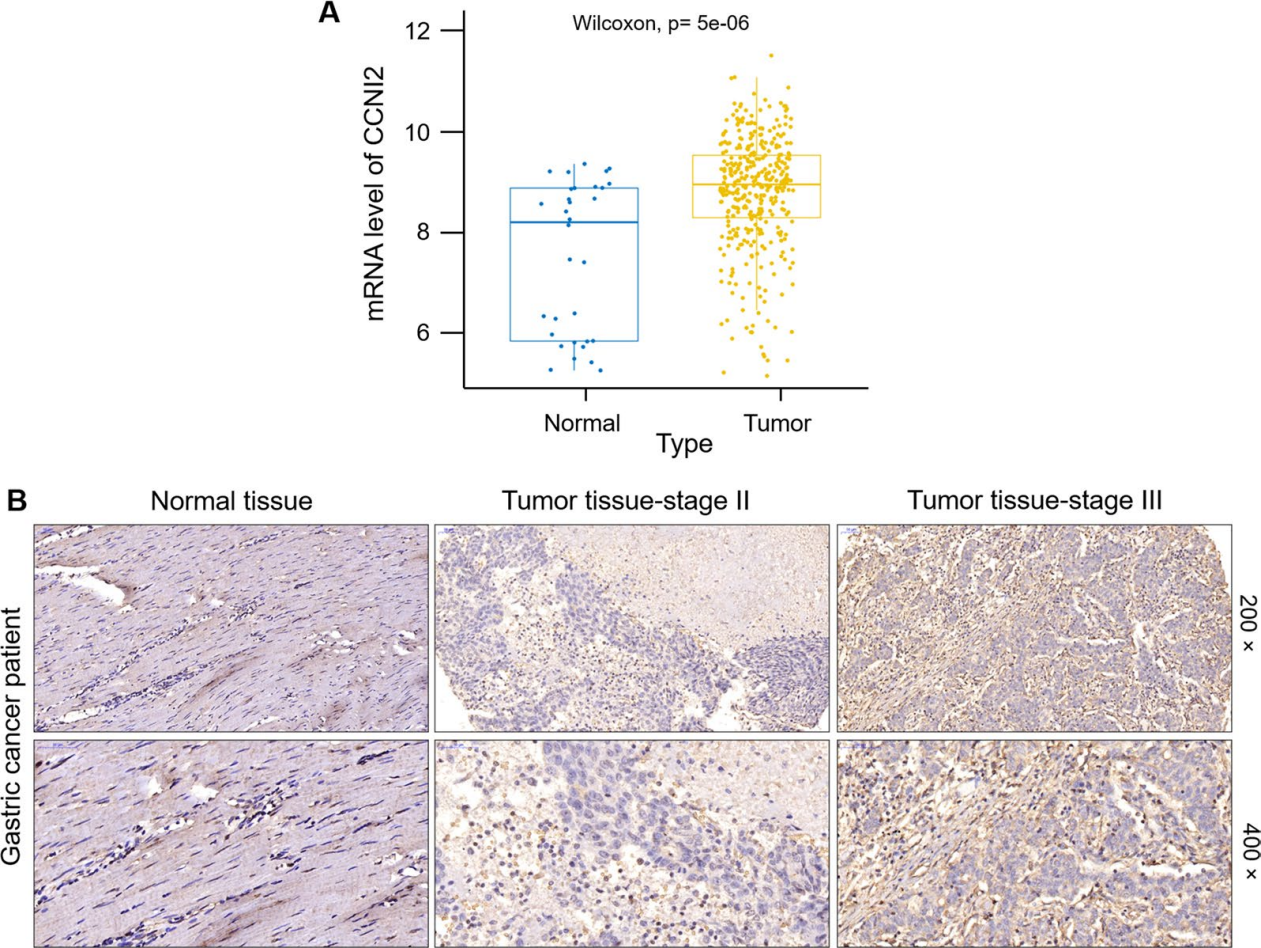
CCNI2 expression is significantly elevated in human gastric cancer. **A** The expression profile of CCNI2 in 407 gastric cancer samples was analyzed based on The Cancer Genome Atlas (TCGA) database. **B** The expression level of CCNI2 in gastric cancer was determined by immunohistochemical staining and representative images were shown. Magnification is 200 and 400

Based on The Cancer Genome Atlas (TCGA) database of 407 gastric cancer samples for expression profile analysis, we found that the expression level of CCNI2 in tumors was significantly higher than that of normal samples (Fig. 1A). The correlation between the expression level of CCNI2 in gastric cancer tissues and clinical prognosis was analyzed (Additional file 1: Fig. S1A). Although the expression level of CCNI2 was not significantly associated with the prognosis of gastric cancer patients, the survival period of gastric cancer patients with high CCNI2 expression was short. Subsequently, we performed TMA and immunohistochemical staining on tissue samples from clinical gastric cancer patients. According to the scoring result of immunohistochemistry, greater than or equal to 4 was considered as CCNI2 high expression. High expression of CCNI2 was observed in 43 of 93 tumor tissue (46.2%) and in 0 of 101 normal tissues (Table 1; Fig. 1B). Consistently, the representative images of immunohistochemical staining showed that the signal intensity of CCNI2 was markedly higher in tumor tissues than in normal tissues (Fig. 1C). To investigate the significance of CCNI2 expression in gastric cancer, we analyzed the correlations of CCNI2 levels with different clinicopathological characteristics. Based on Mann-Whitney U analysis, there was a significant positive correlation between the expression level of CCNI2 and pathological stage (Table 2). In addition, Spearman rank correlation analysis further indicated that as the malignant degree of the tumor deepened, the expression of CCNI2 increased (Table 3). Collectively, CCNI2 was abundantly expressed in gastric cancer and positively correlated with pathological stage.

**Table 1:** Expression patterns in gastric cancer tissues and para-carcinoma tissues revealed in immunohistochemistry analysis

CCNI2 expression	Tumor tissue		Para-carcinoma tissue		p value
	Cases	Percentage (%)	Cases	Percentage (%)	
Low	50	53.8	101	100	< 0.001
High	43	46.2	0	–	

**Table 2:** Relationship between CCNI2 expression and tumor characteristics in patients with gastric cancer

Features	No. of patients	CCNI2 expression		p value
		Low	High	
All patients	93	50	43	
Age (years)				0.331
< 64	44	26	18	
≥ 64	49	24	25	
Gender				0.465
Male	62	35	27	
Female	31	15	16	
T Infiltrate				0.201
T1	6	4	2	
T2	12	9	3	
T3	55	27	28	
T4	20	10	10	
Lymphatic metastasis (N)				0.475
N0	14	12	2	
N1	14	6	8	
N2	20	7	13	
N3	45	25	20	
Stage				0.020
I	5	4	1	
II	22	17	5	
III	63	26	37	
IV	3	3	0	
Tumor size				0.960
< 5 cm	38	20	18	
≥ 5 cm	48	25	23	
Lymph node positive				0.911
≤ 6	47	25	22	
> 6	46	25	21	
Vessel carcinoma embolus				0.879
0	15	8	7	
1	54	30	24	
Nerve tumor infiltrates				0.884
0	21	10	11	
1	18	9	9	
Expression of CD34				0.888
No	10	6	4	
Yes	32	20	12	
Expression of EGFR				0.274
No	69	38	31	
Yes	13	5	8	
Expression of VEGF				0.025*
No	39	26	13	
Yes	43	18	25	
Expression of CDX2				0.202
No	11	8	3	
Yes	73	38	35	
Expression of Her2				0.316
No	64	32	32	
Yes	19	12	7

**Table 3:** Relationship between CCNI2 expression and tumor characteristics in patients with gastric cancer

		CCNI2
Stage	Pearson correlation	0.242
	Significance (double-tailed)	0.020
	N	93

### Downregulation of CCNI2 inhibited proliferation, promoted apoptosis and suppressed migration of gastric cancer cells

**Fig. 2:**
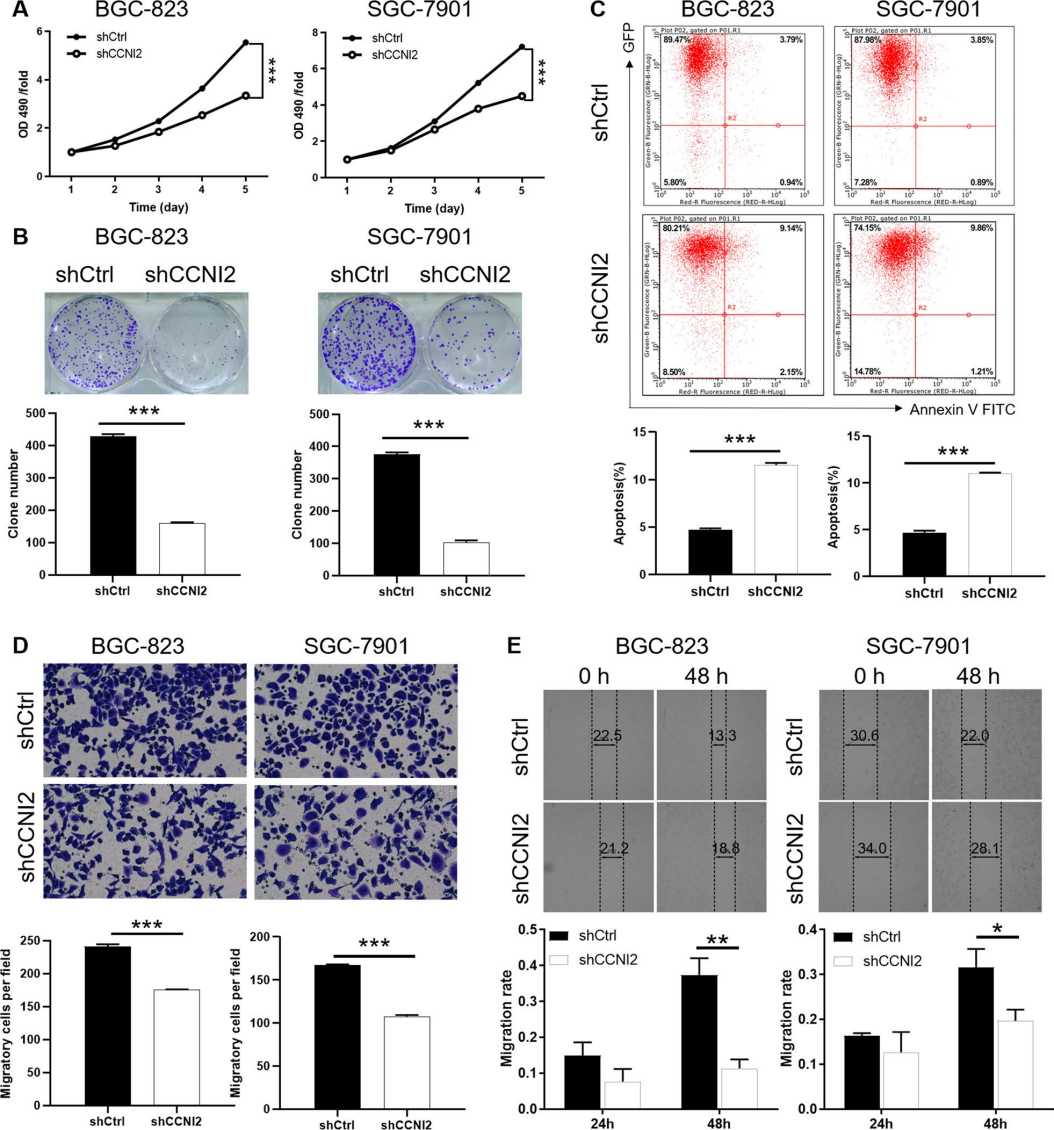
Knockdown of CCNI2 inhibits proliferation, promotes apoptosis and impedes migration of gastric cancer cells. **A** The proliferation of BGC-823 and SGC-7901 cells after knockdown of CCNI2 was measured using MTT assay. **B** Effects of altered CCNI2 expression on the ability of cell clone formation. **C** The effect of CCNI2 knockdown on apoptosis ability was determined by flow cytometry. **D**, **E** The migration of BGC-823 and SGC-7901 cells after knockdown of CCNI2 was measured using Transwell assay (**D**) and wound healing assay (**E**). shCCNI2 indicates CCNI2 knockdown in Gastric cancer cells; shCtrl indicates gastric cancer cells infected with a vector-expressing GFP. The presented results were representative of experiments repeated at least three times. Data was represented as mean ± SD. *P < 0.05, **P < 0.01, ***P < 0.001

To explore the role of CCNI2 in gastric cancer, we determined the effect of CCNI2 downregulation on tumor biological behaviors including proliferation, colony formation, apoptosis, and migration. CCNI2 shRNA (shCCNI2-1) was used in BGC-823 and SGC-7901 cells to knockdown CCNI2 expression (Additional file 1: Fig. S1B). The knockdown efficiency of CCNI2 was assessed at both mRNA and protein levels, indicating that CCNI2 expression was downregulated in BGC-823 and SGC-7901 cells (Additional file 1: Fig. S1C). We first determined the effect of CCNI2 on tumor cell proliferation, suggesting that decrease of CCNI2 expression resulted in reduced proliferation of gastric cancer cells BGC-823 and SGC-7901 (Fig. 2A). Gastric cancer cells with reduced CCNI2 expression produced smaller colonies and reduced numbers (Fig. 2B). Moreover, apoptosis rate in the shCCNI2 group was higher than that in the shCtrl group, indicating that CCNI2 knockdown increased the susceptibility to apoptosis (Fig. 2C). Furthermore, knockdown of CCNI2 in BGC-823 cells upregulated the expression of Bad, BID, and cytoC. On the contrary, the expression levels of HSP60, IGF-II, and sTNF-R1 were downregulated (Additional file 1: Fig. S2A). After CCNI2 expression was reduced, the number of migrating cells was significantly reduced (Fig. 2D). As showed in Fig. 2E, the migration ability of BGC-823 and SGC-7901 with reduced CCNI2 expression was significantly inhibited. Additionally, western blot results showed that CCNI2 knockdown reduced AKT phosphorylation level, downregulated CCND1 and CDK1, and upregulated MAPK9 expression (Additional file 1: Fig. S2B). These results suggested that CCNI2 knockdown slowed the progression of gastric cancer by inhibiting tumor cell proliferation and migration.

### Downregulation of CCNI2 suppressed tumor growth in the mouse xenograft model

**Fig. 3:**
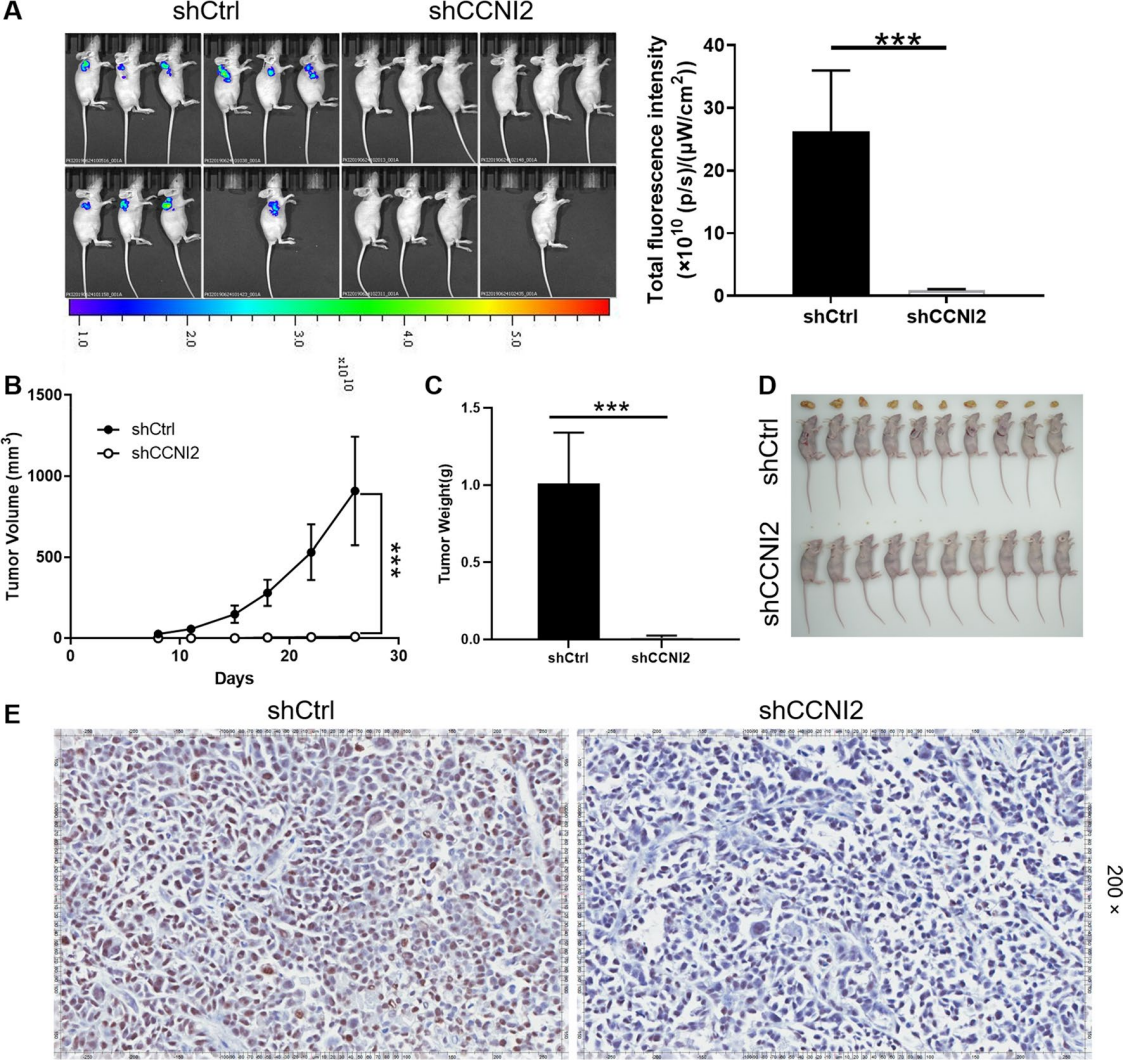
Knockdown of CCNI2 attenuates tumor formation of gastric cancer in vivo. **A** The total bioluminescent intensity was scanned using in vivo imaging system and used as a representation of tumor burden in mice. **B** Post injection of SGC-7901 cells, the tumor volume in mice was measured. (C, D) Mice were sacrificed at day 26 post injection, and the tumor weight was measured (**C**) and photographed (**D**). **E** The expression of Ki67 in mouse tumor tissues was detected by immunohistochemical staining. The presented results were representative of experiments repeated at least three times. Data was represented as mean ± SD. *P < 0.05, **P < 0.01, ***P < 0.001

Mice xenograft models were established in 4-week-old nude mice by injection of CCNI2-knockdown SGC-7901 cells and the tumorigenesis rate was 100% and 50% in shCtrl and shCCNI2 groups, respectively. As illustrated in Fig. 3A, the fluorescence intensity of shCCNI2 group was obviously weaker than that of shCtrl group, suggesting that CCNI2 knockdown inhibited tumor growth. The mice were monitored 26 days after tumor injection, the growth of tumors in shCCNI2 group almost stagnated, while the tumors in the shCtrl group grew rapidly. The largest tumor volume was only 18.23 mm^3^ in the shCCNI2 group and 1306.39 mm^3^ in the shCtrl group (Fig. 3B). Subsequently, the two groups of tumors were weighed. The average weight of the shCCNI2 group (0.012 ± 0.013 g) was significantly lower than that of the shCtrl group (1.011 ± 0.329 g) (Fig. 3C, D). Not surprisingly, the expression of proliferation-related ki67 in mouse tumor tissues showed that the signal of shCCNI2 was weaker than that of shCtrl group (Fig. 3E). Therefore, inhibition of CCNI2 repressed tumor growth in the mouse xenograft model.

### HDGF was the downstream target of CCNI2 in gastric cancer cells

**Fig. 4:**
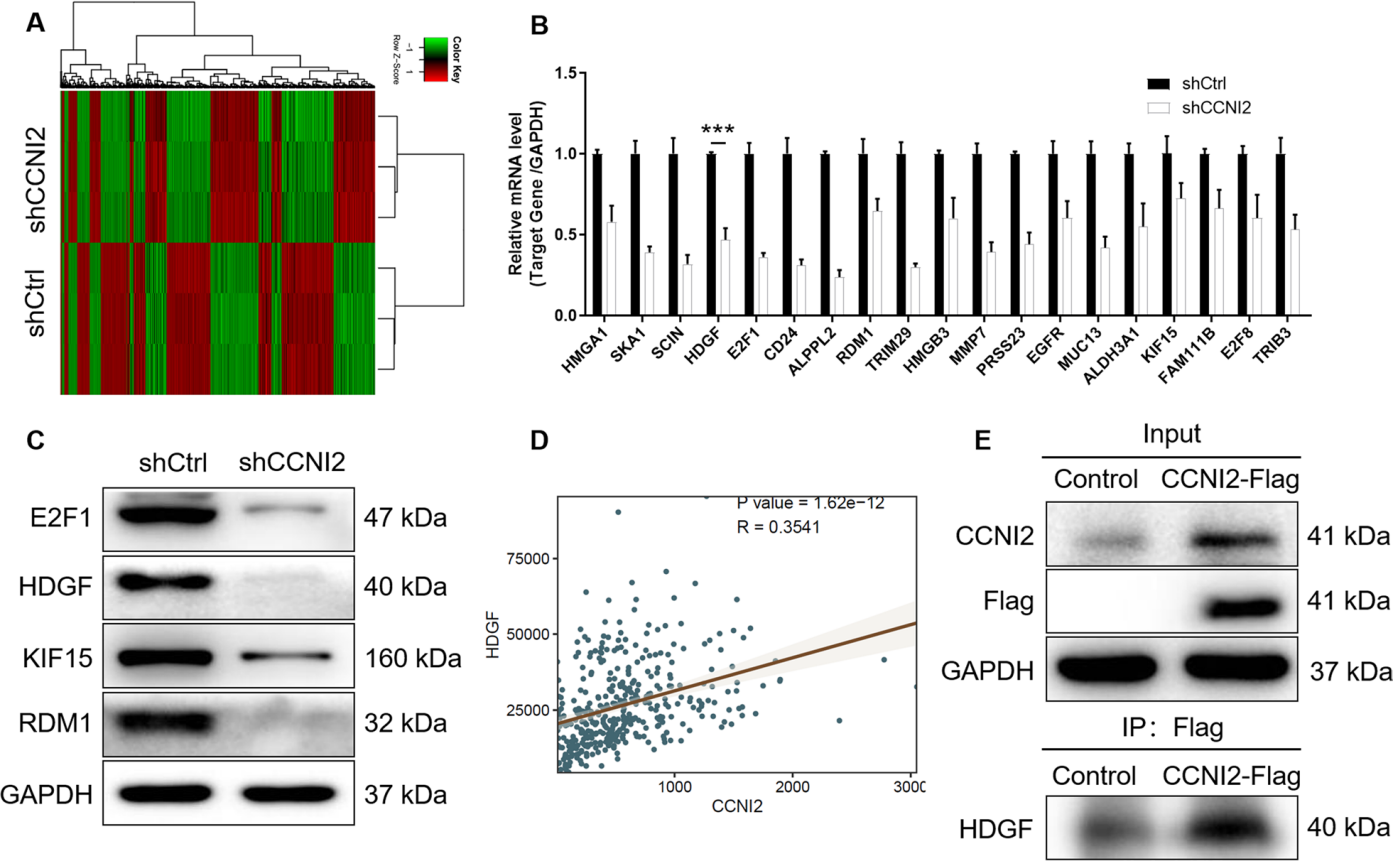
Knockdown of CCNI2 results in the alteration of downstream proteins. **A** The DEGs between shCCNI2 and shCtrl groups of SGC-7901cells was identified. In the heat map of cluster analysis, each column represents a sample and each row represents a differential gene. The red indicates that the gene expression is upregulated, the green indicates that the gene expression is downregulated, the black indicates that the gene expression is not significantly changed, and the gray indicates that the signal strength of the gene is not detected. **B**, **C** The expression of several selected DEGs of SGC-7901 cells after knockdown of CCNI2 was measured by qPCR (B) and western blot (**C**). **D** Pearson correlation analysis indicated a significant positive correlation between CCNI2 and HDGF expression. **E** Proteins interaction between CCNI2 and CDK1 was determined by Co-IP assay. The presented results were representative of experiments repeated at least three times. Data was represented as mean ± SD. *P < 0.05, **P < 0.01, ***P < 0.001

In order to clarify the role of CCNI2 in gastric cancer, its potential mechanism was initially explored. RNA sequencing results showed that CCNI2 knockdown resulted in abnormal expression of DEGs, of which 1157 genes were upregulated and 1303 genes were downregulated. Figure 4A is a heat map of hierarchical clustering of shCCNI2 and shCtrl two samples using |Fold Change|≥1.3 and FDR<0.05 as standard screening of differential gene expression profiles. Additionally, the interaction network between CCNI2 and DEGs was further analyzed based on IPA (Additional file 1: Fig. S3). The most significant DEGs were screened by PCR (Fig. 4B) and western blot (Fig. 4C), the results indicated that HDGF was downregulated after CCNI2 knockdown in SGC-7901 cells. Additionally, Pearson correlation analysis indicated a significant positive correlation between CCNI2 and HDGF expression (Fig. 4D). As illustrated in Fig. 4E, the Co-IP assay validated that there was protein interaction between CCNI2 and HDGF in SGC-7901 cells. Therefore, we preliminarily determined that HDGF was regulated by CCNI2 in gastric cancer and may be a downstream target of CCNI2.

### Knockdown of CCNI2 alleviates the promoting effects of HDGF overexpression in gastric cancer cells

**Fig. 5:**
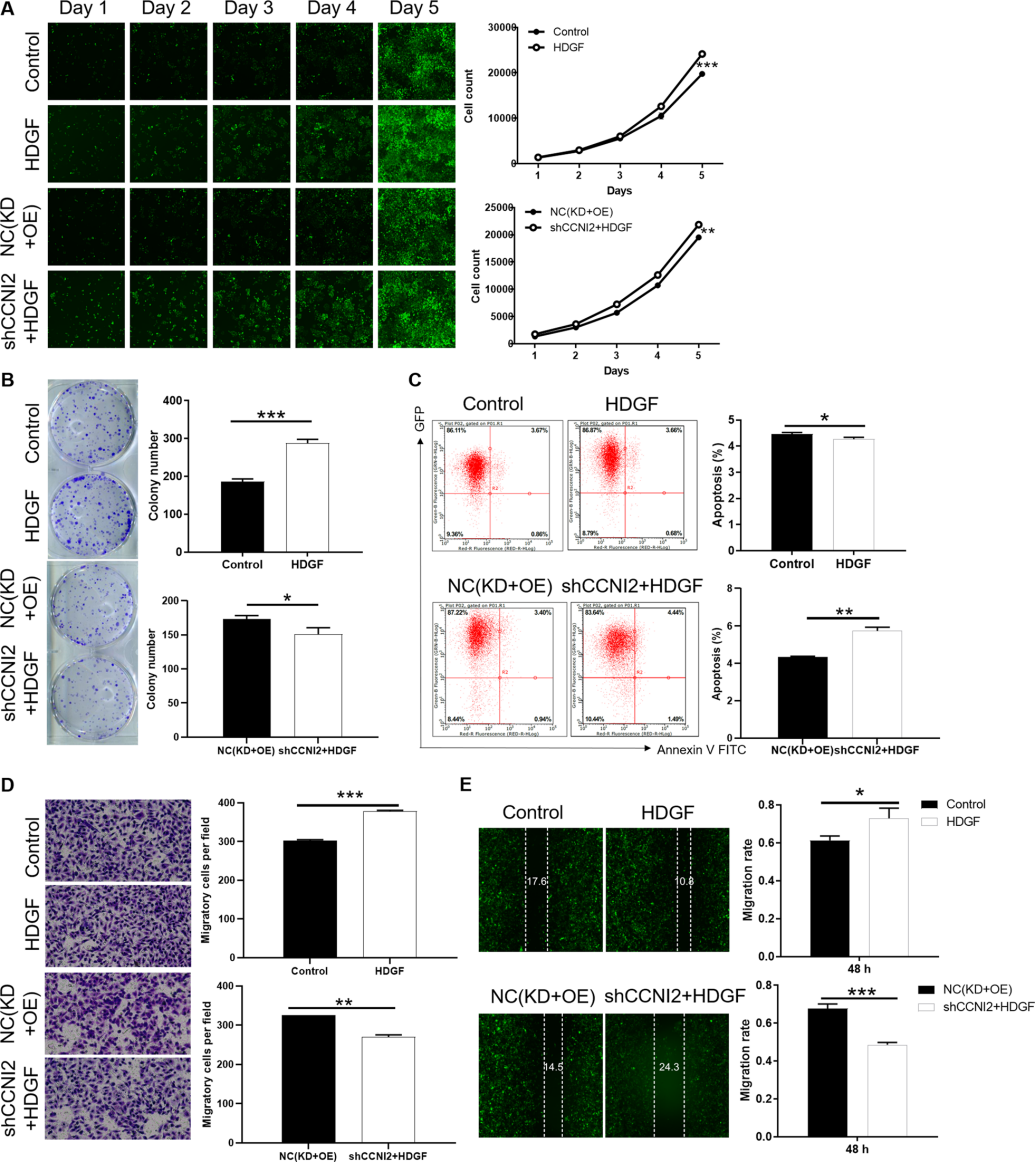
Knockdown of CCNI2 alleviates the promoting role of HDGF overexpression in gastric cancer cells. Detection of alteration in proliferation (**A**), clone formation (**B**), apoptosis (**C**) and migration (**D**, **E**) after lentivirus HDGF, and shCCNI2+HDGF transfected SGC-7901cells. HDGF indicates HDGF overexpression in SGC-7901cells; Control indicates SGC-7901 cells infected with an empty vector LV-003, as negative control; shCCNI2+HDGF indicates simultaneously downregulated CCNI2 and upregulated HDGF in SGC-7901cells; NC(KD+OE) indicates SGC-7901 cells infected with empty vector LV-003 and BR-V108, as negative control. The presented results were representative of experiments repeated at least three times. Data was represented as mean ± SD. *P < 0.05, **P < 0.01, ***P < 0.001

Since there was a relationship between CCNI2 and HDGF, their biological functions in gastric cancer cell lines deserved further investigation. SGC-7901 cells with high expression of HDGF were used to reveal the alterations of biological phenotypes. As showed in Fig. 5A, SGC-7901 cells with high HDGF expression showed a proliferation promoting effect. Meanwhile, the high expression of HDGF produced larger and more cell clones compared with the control group (Fig. 5B). Moreover, the apoptotic ability of SGC-7901 cells with high HDGF expression was reduced (Fig. 5C). The migration ability of SGC-7901 cells with HDGF overexpression was stronger than that of the control group (Fig. 5D, E). As a consequence, we clarified that HDGF can promote the malignant progression of gastric cancer. Furthermore, the SGC-7901 cells with both CCNI2 downregulation and HDGF upregulation (shCCNI2+ HDGF) were established. Interestingly, shCCNI2+HDGF group could slow down the malignant progression of HDGF overexpression group in SGC-7901 cells, which was characterized by reducing proliferation (Fig. 5A), forming fewer clones (Fig. 5B), increasing apoptosis (Fig. 5C) and inhibiting migration (Fig. 5D, E). Taken together, the loss/gain-of-function assays demonstrated that knockdown of CCNI2 could alleviate the promoting effects of HDGF overexpression in SGC-7901 cells.

## Discussion

In view of the fact that gastric cancer is a multi-stage disease process, characterized by the gradual development of various gene expression mutations and epigenetic alterations [2]. A major breakthrough of this study is the identification of promoting effect of CCNI2 in human gastric cancer. We found that CCNI2 was abundantly expressed in gastric cancer and was positively correlated with pathological stage. Additionally, inhibition of CCNI2 can slow down the malignant progression of gastric cancer by inhibiting tumor cell proliferation, increasing the susceptibility to apoptosis and suppressing migration. The malignant transformation of normal cells is caused in part by aberrant gene expression disrupting the regulation of cell proliferation, senescence and apoptosis [23]. Previous study clarified those alterations in apoptosis were usually related to the occurrence and development of tumors, involving a series of cell signals [24]. The present study revealed that knockdown of CCNI2 in BGC-823 cells upregulated the expression of cytoC (cytochrome c), Bad (Bcl-2 antagonist of cell death), and BID. On the contrary, the expression levels of HSP60 (heat shock protein 60), IGF-II (Insulin-like growth factor II), and sTNF-R1 (tumor necrosis factor (TNF) and its soluble receptors type 1) were downregulated. Apoptosis is performed by caspases, a subfamily of cysteine proteases. One of the main caspase activation pathways is the activation of cytoC, which in turn induces a series of biochemical reactions, leading to caspase activation and subsequent cell death [25]. Pro-apoptotic factor Bad-mediated apoptotic pathway was associated with human cancer development [26]. Bid is an abundant pro-apoptotic protein of the Bcl-2 family that is crucial for death receptor-mediated apoptosis in many cell systems [27]. Furthermore, the reduction of HSP60 expression can lead to cell apoptosis, which plays a key regulatory role in cell apoptosis [28]. IGF-II is an anti-apoptotic protein, which is highly expressed in β cells during development and stimulates cell survival and proliferation [29]. sTNF-R1 as a key mediator between apoptosis and cancer cell progression [30]. On this basis, the alterations in key apoptotic factors after CCNI2 knockdown were consistent with the apoptotic phenotypes of gastric cancer cells. However, the molecular mechanism that CCNI2 regulated apoptosis in gastric cancer need more exploration.

Studies have shown that phosphatidylinositol 3-kinase (PI3K)/AKT signaling pathway is involved in a variety of carcinogenic processes, including cell proliferation, growth, survival, apoptosis and migration [31]. CCND1 (Cyclin D1) overexpression correlated with poor tumor differentiation and prognosis in gastric cancer [32]. In this study, western blot analysis found that CCNI2 knockdown resulted in reduced AKT phosphorylation level, downregulated CCND1 and CDK1, upregulated MAPK9 expression in gastric cancer cells.

Additionally, we studied the regulation mechanism of CCNI2 on gastric cancer and found that HDGF may be the downstream of CCNI2. Transcription factor hepatoma-derived growth factor (HDGF) is an acidic heparin-binding growth factor, originally isolated from the culture medium of human hepatocellular carcinoma cell line HuH-7 [33]. Previous studies have shown that HDGF can be translocated into the nucleus and act as a direct DNA-binding protein, possibly as both cytokine and transcription factor [34–37]. HDGF is endogenously expressed in endothelial cells and can induce exogenous angiogenesis [38]. Moreover, HDGF plays an important role in cell cycle, apoptosis, cytokine signal transduction and metabolism of embryonic stem cells [39]. HDGF is involved in tumor-related events such as tumorigenesis, metastasis, and angiogenesis [40]. For instance, HDGF promotes growth and metastasis of hepatocellular carcinoma cells [41]. Studies have shown that glioblastoma stem cell-like cells can express HDGF to directly induce tumor angiogenesis [42]. In addition, downregulation of HDGF inhibits the tumorigenesis of bladder cancer cells by inactivating the PI3K/AKT signaling pathway [43]. Importantly, HDGF participates in Helicobacter Pylori-induced neutrophils recruitment, gastritis and gastric carcinogenesis [44].

## Conclusions

Our study found that HDGF overexpression exhibited a significant promotion in the progression of gastric cancer cells. Besides, knockdown of CCNI2 could alleviate the promoting role of HDGF overexpression in gastric cancer cells. Thus, CCNI2 promote the development and progression of gastric cancer through HDGF, which may be a therapeutic target for gastric cancer.

## Supplementary Information

**Additional file 1.** Additional figures and tables.

## Data Availability

All data generated or analysed during this study are included in this published article and its additional files.
