# Supplementary material for: CCNI2 promotes the progression of human gastric cancer through HDGF
Source: Cancer Cell Int. 2021 Dec 11;21:661. doi: 10.1186/s12935-021-02352-6 (PMC8665640; doi:10.1186/s12935-021-02352-6)
Supplement: Supplementary file 1 — Additional file 1. Additional figures and tables. [file 12935_2021_2352_MOESM1_ESM.docx]

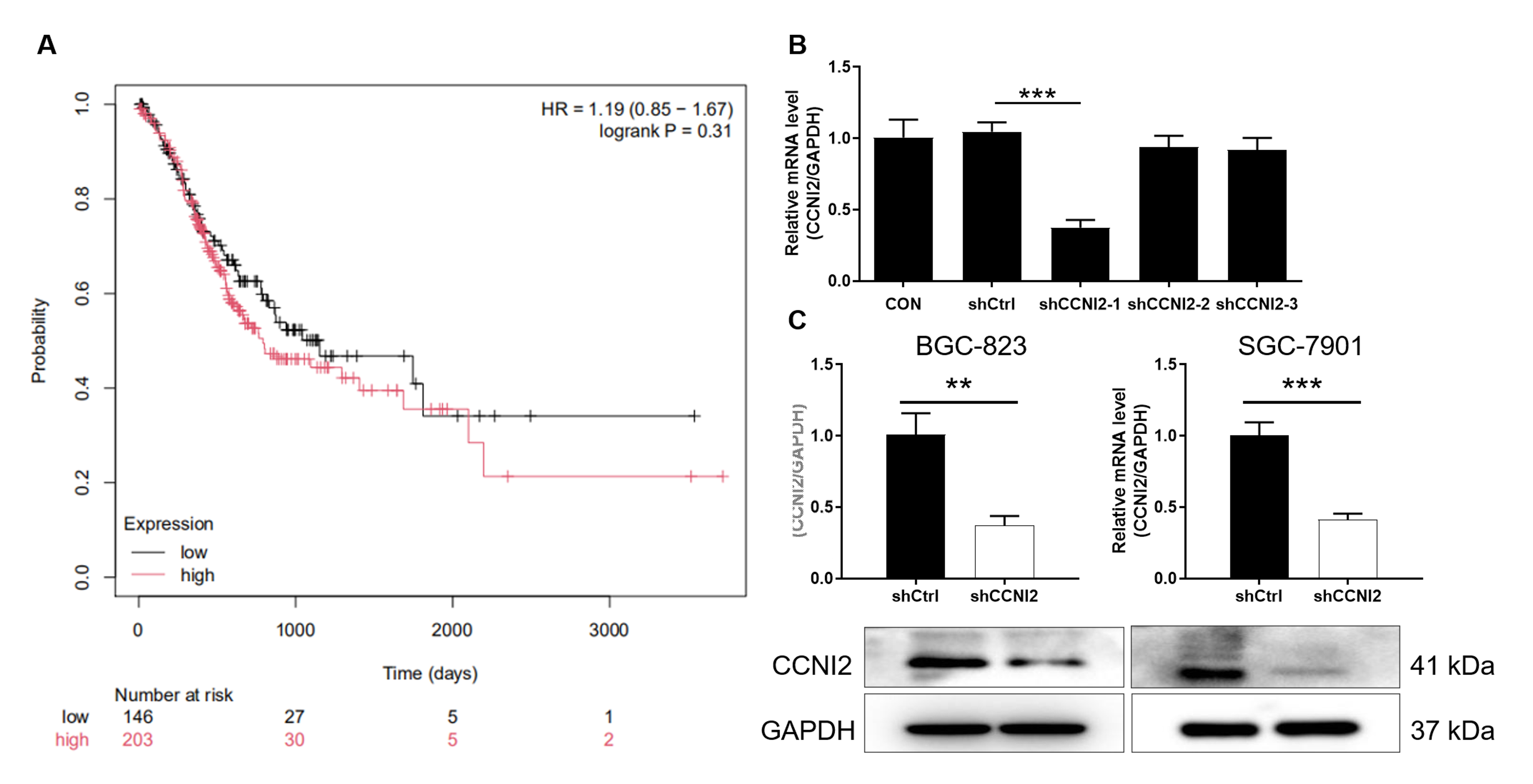


**Fig. S1** (A) The correlation between the expression level of CCNI2 in gastric cancer tissues and clinical prognosis was analyzed. (B) The expression level of CCNI2 was detected in 3 different shRNAs against CCNI2 sequences. (C) The specificity and validity of the lentivirus-mediated shRNA knockdown of CCNI2 expression was verified by qPCR and WB. The data were presented as the mean ± SD (n ≥ 3), *P<0.05, **P<0.01, ***P<0.001.


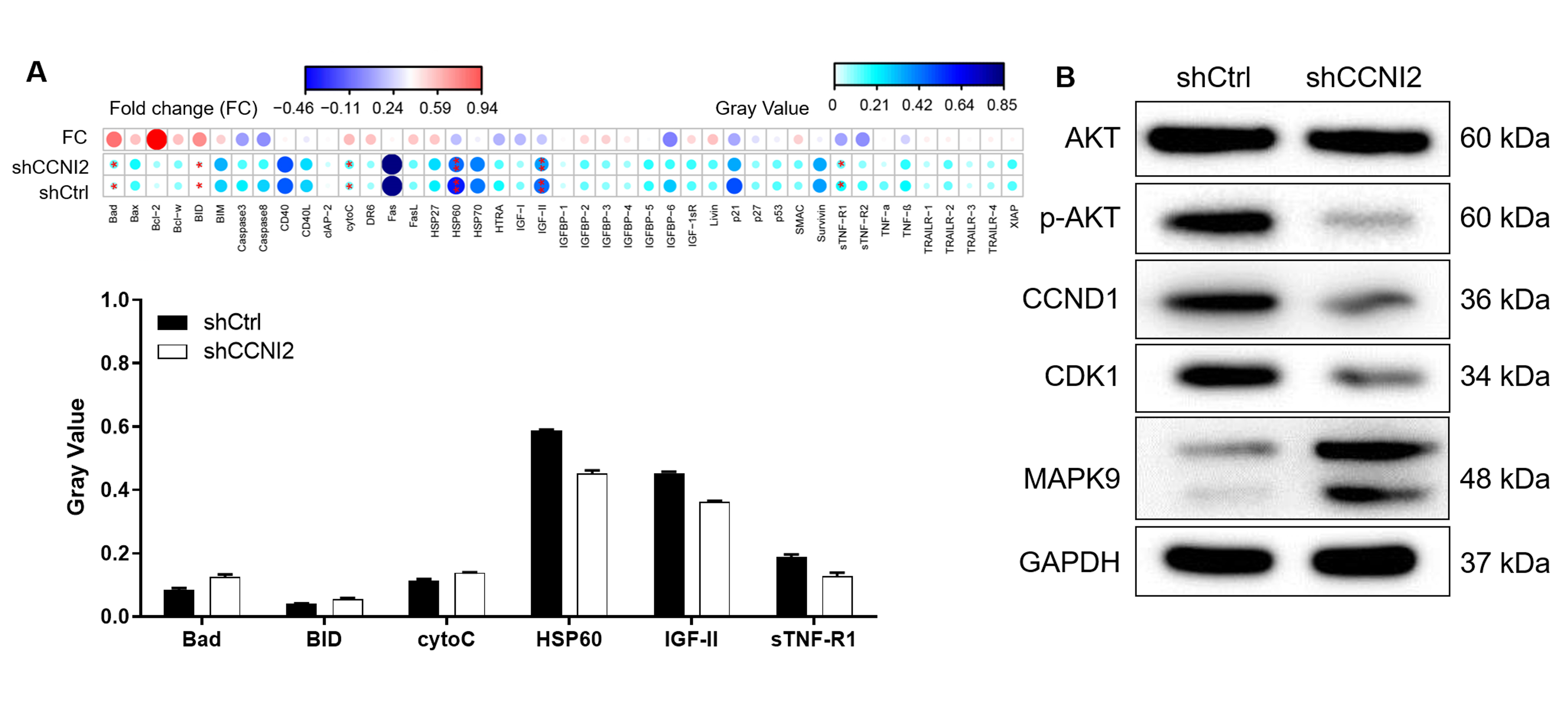


**Fig. S2** (A) The expression of 43 apoptosis-related regulators of BGC-823 cells after knockdown of CCNI2 was detected using apoptotic antibody array kit (n = 2). (B) The protein expression of AKT, p-AKT, CCND1 and PIK3CA of BGC-823 cells after knockdown of CCNI2 was measured by WB.

**
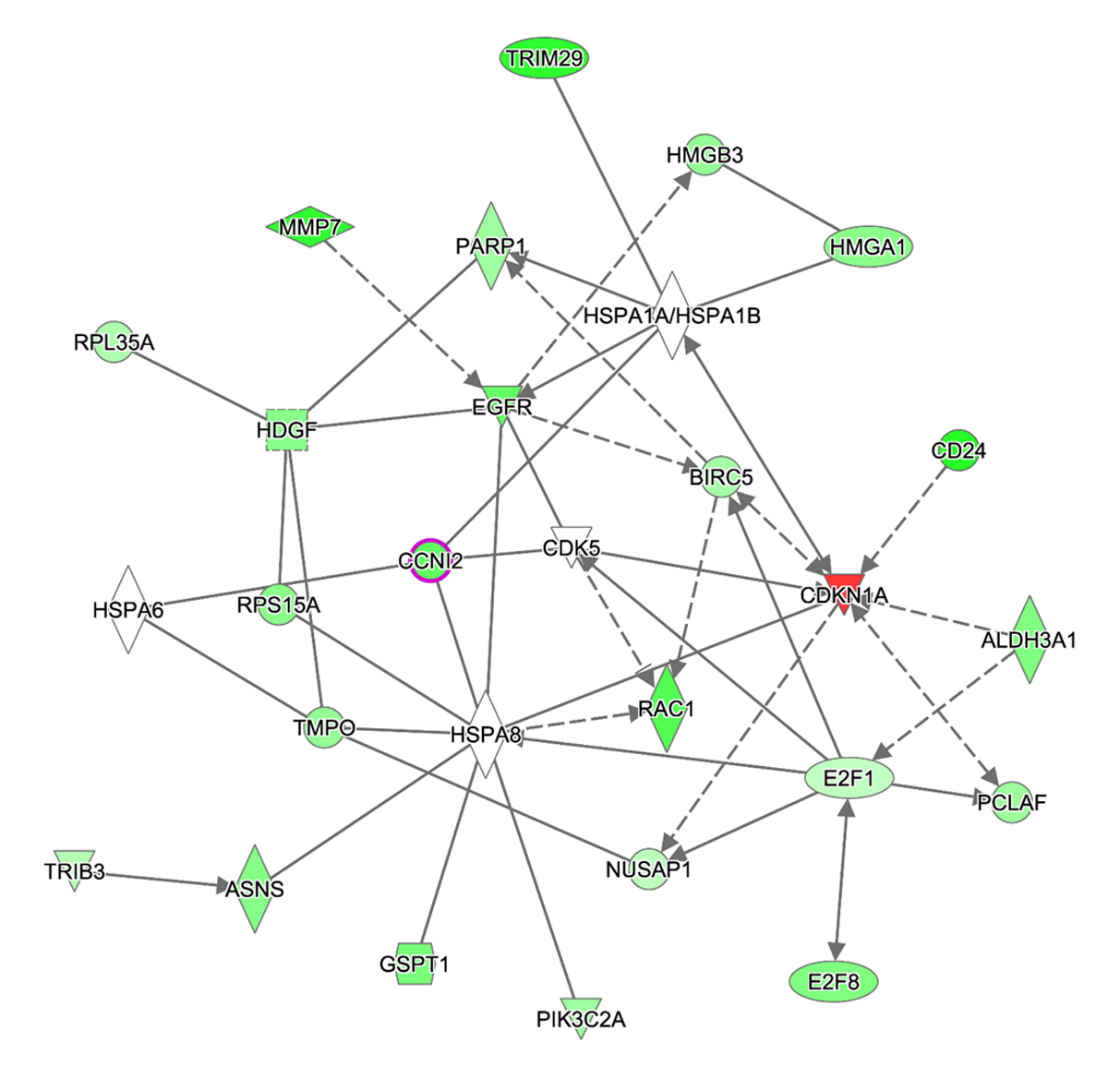
**

**Fig. S3** Interaction network diagram among DEGs was analyzed after knockdown of CCNI2 in BGC-823 cells by IPA.

Table S1. Primer sequence for PCR.

| Gene | Upstream primer sequence | Downstream primer sequence | Amplified fragment size  (bp) |
| --- | --- | --- | --- |
| GAPDH | AAATACCAGCCCAGGAAGCA | AAACTGAGGCTCTGGGGACA | 121 |
| CCNI2 | CCAGGGAGTATGAATGAATGTT | TTGGGATAAGCCTGGGAAGTT | 209 |
| HMGA1 | AGCCCAGCGAAGTGCCAACA | CCTCCTCTTCCTCCTTCTCCAGTTTT | 150 |
| SKA1 | TCCCATTTGCCTCAAGTAACAG | GGAGGCTTCTTTACGGGTTC | 107 |
| SCIN | ACAACATCTGCGTTCCTGACTG | GCTCTTTGCCTTGGGAGACT | 85 |
| HDGF | AGCCCAACAAGAGGAAAGGG | ACTGATAGCCGGAAGCCTTG | 81 |
| E2F1 | CACTTTCGGCCCTTTTGCTC | GTGCTCTCACCGTCCTACAC | 128 |
| CD24 | TGCTCCTACCCACGCAGATT | GGTGGTGGCATTAGTTGGATTT | 113 |
| CDKN1A | TCTTGTACCCTTGTGCCTCG | GAAATCTGTCATGCTGGTCTGC | 105 |
| ALPPL2 | AAGCAGGAAAGTCAGTGGGAGTGGTAA | AGGCACGTCGGCATCCGAGTA | 113 |
| RDM1 | GCCCATCCTGGTTTCTATGC | GGCTTGATGTTGAACTGCCTTA | 150 |
| TRIM29 | CAAGGAGACCACCCAGAAGAA | GGGCAGGTCATTGTCAGAGTT | 112 |
| HMGB3 | ACCGTCTGGATTCTTCCTGTT | GATGTAAGGCTGCTTTTCACTG | 142 |
| MMP7 | GACTTCCAAAGTGGTCACCTACA | CAGTTCCCCATACAACTTTCCT | 143 |
| PRSS23 | GAAGGAAGCGGCAGATTTATGG | TGATGTTGAGAAAGGGTAGTTGAGC | 86 |
| EGFR | ATGAGGACATAACCAGCCACC | AGGCACGAGTAACAAGCTCAC | 177 |
| MUC13 | GTTGCTTCCAGTCTCAAGTGTCC | CAGTTCCCATTAGCATCTTCCTG | 128 |
| ALDH3A1 | TGGAGCTGGGAGGGAAGAGTC | GAGGGGTCACAGAGGATGTAGTCA | 139 |
| KIF15 | CTCTCACAGTTGAATGTCCTTG | CTCCTTGTCAGCAGAATGAAG | 114 |
| FAM111B | CCAGACAATTCCCAGGATTAGA | TAGCATACCGCCTACCCAGA | 110 |
| E2F8 | TGACGAAGTGGCAGAGGAAC | CATCATAATCTGCTCGGCGTA | 196 |
| TRIB3 | AGCGGTTGGAGTTGGATGA | TTGCACGATCTGGAGCAGTAG | 129 |

Table S2. Antibodies used in western blot and Co-IP.

| Primary antibodies | Dilution in WB | Source species | Company | Catalog No. |
| --- | --- | --- | --- | --- |
| CCNI2 | 1:1000 | Rabbit | Abcam | ab97767 |
| AKT | 1:1000 | Rabbit | CST | 4685 |
| p-AKT | 1:500 | Rabbit | R&D | AF887-sp |
| CCND1 | 1:1000 | Rabbit | CST | 2978 |
| CDK1 | 1:3000 | Rabbit | Abcam | ab133327 |
| MAPK9 | 1:3000 | Rabbit | Abcam | ab76125 |
| E2F1 | 1:1000 | Rabbit | Abcam | ab179445 |
| HDGF | 1:1000 | Rabbit | Abcam | ab128921 |
| KIF15 | 1:1000 | Rabbit | fine test | FNab04551 |
| RDM1 | 1:1000 | Rabbit | biorbyt | orb352658 |
| GAPDH | 1:3000 | Rabbit | Bioworld | AP0063 |
| Primary antibodies | Dilution in Co-IP | Source species | Company | Catalog No. |
| DYKDDDDK Tag | 1:50/1:1000 | Rabbit | CST | 14793S |
| CCNI2 | 1:2000 | Rabbit | Abcam | ab97767 |
| HDGF | 1:1000 | Rabbit | Abcam | ab128921 |
| GAPDH | 1:30000 | Rabbit | Bioworld | AP0063 |
| Secondary antibody | Dilution |  | Company | Catalog No. |
| HRP Goat Anti-Rabbit IgG (western blot) | 1:3000 |  | Beyotime | A0208 |
| HRP Goat Anti-Rabbit IgG (Co-IP) | 1:3000 |  | Beyotime | A0216 |
